# Decoding Multiple Sound Categories in the Human Temporal Cortex Using High Resolution fMRI

**DOI:** 10.1371/journal.pone.0117303

**Published:** 2015-02-18

**Authors:** Fengqing Zhang, Ji-Ping Wang, Jieun Kim, Todd Parrish, Patrick C. M. Wong

**Affiliations:** 1 Department of Statistics, Northwestern University, Evanston, Illinois, United States of America; 2 Department of Psychology, Drexel University, Philadelphia, Pennsylvania, United States of America; 3 The Roxelyn and Richard Pepper Department of Communication Science and Disorders, Northwestern University, Evanston, Illinois, United States of America; 4 Department of Radiology, Feinberg School of Medicine, Northwestern University, Chicago, Illinois, United States of America; 5 Department of Linguistics & Modern Languages, The Chinese University of Hong Kong, Shatin, Hong Kong, PRC; 6 Department of Otolaryngology—Head & Neck Surgery, Northwestern University, Chicago, Illinois, United States of America; Harvard Medical School/Massachusetts General Hospital, UNITED STATES

## Abstract

Perception of sound categories is an important aspect of auditory perception. The extent to which the brain’s representation of sound categories is encoded in specialized subregions or distributed across the auditory cortex remains unclear. Recent studies using multivariate pattern analysis (MVPA) of brain activations have provided important insights into how the brain decodes perceptual information. In the large existing literature on brain decoding using MVPA methods, relatively few studies have been conducted on multi-class categorization in the auditory domain. Here, we investigated the representation and processing of auditory categories within the human temporal cortex using high resolution fMRI and MVPA methods. More importantly, we considered decoding multiple sound categories simultaneously through multi-class support vector machine-recursive feature elimination (MSVM-RFE) as our MVPA tool. Results show that for all classifications the model MSVM-RFE was able to learn the functional relation between the multiple sound categories and the corresponding evoked spatial patterns and classify the unlabeled sound-evoked patterns significantly above chance. This indicates the feasibility of decoding multiple sound categories not only within but across subjects. However, the across-subject variation affects classification performance more than the within-subject variation, as the across-subject analysis has significantly lower classification accuracies. Sound category-selective brain maps were identified based on multi-class classification and revealed distributed patterns of brain activity in the superior temporal gyrus and the middle temporal gyrus. This is in accordance with previous studies, indicating that information in the spatially distributed patterns may reflect a more abstract perceptual level of representation of sound categories. Further, we show that the across-subject classification performance can be significantly improved by averaging the fMRI images over items, because the irrelevant variations between different items of the same sound category are reduced and in turn the proportion of signals relevant to sound categorization increases.

## Introduction

Perception of sound categories is fundamental to our everyday life. One of the core aspects of auditory perception is to abstract discrete categories from continuous physical features when stimuli are grouped into distinct but meaningful categories [1]. Categorization requires minimizing within-category and maximizing between-category differences, which is a more abstract representation of sound similarity. By doing so, continuous physical variations between stimuli are overruled such that seemingly distinct stimuli may be considered as the same category. Several attempts have been made to investigate the brain’s representation of sound categories [1–3]. Several studies supported a hierarchically organized object-processing pathway along anteroventral auditory cortex [3, 4], while others stressed the importance of distributed representations of auditory objects [2, 5]. The extent to which the representation and processing of auditory categories are encoded in specialized subregions or distributed across the auditory cortex remains unclear.

Recent functional magnetic resonance imaging (fMRI) studies have applied different statistical methods to brain decoding and have provided important insights into how the brain decodes perceptual information [5–11]. Multivariate pattern analysis (MVPA) has drawn increasing attention in fMRI studies [10–13]. Whereas traditional univariate approaches examine only one location (voxel) at a time, MVPA extracts information from many brain locations (voxels) simultaneously, thereby examining the full spatial pattern of brain responses. In the study of the representation of visual-object categories in the ventral temporal cortex, it has been demonstrated that MVPA is sensitive to changes in distributed activation patterns in absence of changes in overall activation level [14]. MVPA approach has been taken to reveal subtle differences in overlapping sound representations [2, 5]. However, these studies have either largely focused on binary classification, or they tended to examine visual perception [5–9]. In the large existing literature on brain decoding using MVPA methods, there is a relative lack of studies on multi-class categorization in the auditory domain. The rich variety of auditory categories in real life creates a need for decoding strategies to consider multiple categories simultaneously. Multi-class MVPA models permit the use of more than two categories. Hence, in this work we study decoding multiple sound categories simultaneously in the human temporal cortex using high resolution fMRI and multi-class MVPA models.

In fMRI studies of auditory functions, there are data analysis challenges because the sound signal is continuous and changes with time. The recognition of sound objects requires continuous listening and continuous processing of its cognitive meaning. This may cause the hemodynamic responses to be less informative for discriminating between different auditory stimuli. In addition, McDermott *et al*. showed that both visual and auditory processing require summary statistics but visual processing is spatial while auditory processing is temporal [15–17]. Summary statistical measures in vision occur over spatial regions of the visual field while auditory effects involve pooling information over time [15, 18]. One fundamental study in speech perception combined fMRI technology with a statistical pattern recognition method (support vector machine-recursive feature elimination, SVM-RFE) to demonstrate the feasibility of decoding speech content and speaker identity [5]. Their analysis, however, was restricted to binary classification and did not consider decoding multiple auditory objects simultaneously. In everyday life a person experiences a large number of auditory objects, creating a need for decoding strategies to generalize across multiple auditory objects and different individuals. In most human decoding studies to date, decoding algorithms have been trained on each participant individually and/or for a fixed set of mental states, which is a highly simplified situation compared to real-world applications [5, 6, 9, 10]. The extent to which MVPA decoding models can be generalized to different circumstances, such as multiple sound categories, different subjects, and different sound exemplars within the same sound category, is currently not well understood [9, 10].

In this study, we investigated the representation and processing of auditory categories within the human temporal cortex using high resolution fMRI and MVPA methods. More importantly, we considered decoding multiple sound categories simultaneously through multi-class classifiers as our MVPA tools. We first built multi-class MVPA models for each subject separately to predict which of seven sound categories the subject heard. This within-subject analysis allowed us to explore spatially distinct patterns of sound category selective activity in the human temporal cortex. We then built multi-class (all seven sound categories) classifiers across subjects. In this procedure, a classifier learned to distinguish the neural patterns evoked by each sound category based on the data from a subgroup of the subjects and was then tested on the data from a subject that was not part of that subgroup. In this across-subject analysis, we asked whether there are consistent brain activity patterns across subjects and how results from pattern analysis are generalized across different subjects. Categorical perception of sounds requires a more abstract representation of sound similarity. The formation of the category boundary requires perceptual invariance of sets of objects classified as belonging to the same category and ignorance of irrelevant differences in some aspects. Not all sound exemplars are equivalent in regard to category membership. Therefore, we explored the effects of category membership on brain activity patterns and decoding accuracy.

## Materials and Methods

### Ethics statement

The study was approved by Northwestern University’s Institutional Review Board and written informed consent was obtained from all participants.

### Participants

Six right-handed monolingual English speakers (mean age, 22.5 years; 4 female) from Northwestern University were recruited for the functional imaging study. They had no history of neurological disorders and reported normal hearing. Similar sample size was used in several other high resolution fMRI studies for examining human brain decoding [1, 2, 5, 19, 20].

### Stimuli

Seven sound categories were used, including English speech (EN), non-English speech (NE), non-speech vocal (VC), animal (AN), mechanical (MC), music (MS), and nature sounds (NT). These were cognitive categories, which are abstract and likely differ along multiple dimensions, rather than perceptual categories which, via identification and discrimination tasks, show clear hallmarks of categorical perception (such as speech consonants). Each category consisted of six different sound items (sound exemplars), i.e. a total of 42 tokens (stimuli) were used (Table 1). Stimuli were one second in duration, taken from websites (AN, MC, MS, and NT) and original recordings (EN, NE, and VC). All stimuli were resampled to 44.1 kHz and normalized to the same intensity level and fundamental frequency using the Praat and Level 16 software [21, 22].

**Table 1:** Stimulus categories and the quality score (QS) of sound exemplars averaged within each category.

**Item #**	**English**	**Non-English**	**Vocal**	**Animal**	**Mechanical**	**Music**	**Nature**
**1**	Bill	Bengali_M	Pain	Duck	Gunshot	Violin	Ocean
**2**	Dan	Bulgarian_F	Baby cry	Bird	Clock_tick	Guitar	Water
**3**	Josh	Chinese_M	Cough	Cow	Car_start	Drum	Hurricane
**4**	Jenny	Hindi_F	Giggle	Frog	Phone	Cello	Brook
**5**	Ann	Kazakhstan_M	Snore	Horse	Toilet	Piano	River
**6**	Mary	Chinese_F	Yawn	Lamb	Typing	Saxophone	Wind
**QS**	4.6	4.6	4.6	4.5	4.5	5.0	4.4

To ensure sound quality and identification accuracy, a behavioral pilot study was done on ten volunteers who did not participate in the scanning sessions. They were asked to describe what they heard in a short sentence and to score the sound quality on a scale from one (the worst) to seven (the best) after listening to each stimulus. All subjects, except one who could not recognize most sounds, were able to correctly identify individual stimuli. Results from this pilot study were used for the process of stimuli construction and selection. As shown in Table 1, the averaged sound quality scores for each category ranged between 4.4 and 5.0, indicating adequate quality of the sound stimuli. In addition, after the scan, all subjects who participated in the fMRI scanning were asked and confirmed that they were able to accurately categorize individual stimuli.

### Experimental design and fMRI acquisition

A slow event-related design with an average inter-stimulus-interval (ISI) of 15 seconds was adopted. A slow event-related design with a long ISI permits time-course estimates of the BOLD response to single presentations of sounds by measuring the impulse response of the stimuli. Each of the seven functional runs consisted of 42 stimulus trials in which each sound stimulus was presented only once. The sequence of stimuli was pseudo-randomized across runs.

Brain imaging was performed at the Center for Advanced MRI in the Department of Radiology at Northwestern University using a Siemens 3T Trio. For each subject, seven high-spatial resolution (1.7 mm × 1.7 mm × 2 mm) functional runs (215 volumes per run = 5 volumes x 43 trials, 10 minutes 45seconds = 15 seconds x 43 trials) were collected for two days using a standard echo-planar-imaging (EPI) sequence (TR = 3s, TA = 2s, TE = 20ms, 1 trial = 15 seconds = 5 repetitions of 2-second scan and 1-second silence). The initial 5 volumes of each run were discarded to allow the MR signal to reach equilibrium. Each volume consisted of 32 contiguous slices without a gap, covering the entire temporal lobe. During the functional runs, subjects listened to the stimuli that were presented via MR compatible insert earphones (Sensimetrics, Inc, Boston) in the 1 second silent interval between two volume acquisitions. The sound stimulus was presented in one of these five 1-second silences. The presentation of the stimuli in a silent gap resulted in a clear perception of the acoustic stimuli and separation from the scanning noise. An anatomical image covering the whole brain was also obtained using a high-resolution T1-weighted sequence (1 mm × 1 mm × 1 mm).

### Data pre-processing

Functional and anatomical images were analyzed with AFNI [23]. Functional images were pre-processed for slice timing correction, rigid body motion correction, linear trend removal, temporal high-pass filtering, coregistration to individual structural images, and normalization of anatomical and functional data to Talairach space. No spatial smoothing was applied to preserve the fine-grained spatial information from the high resolution functional MRI.

At each voxel, the stimulus response was obtained by a general linear model (GLM) with one predictor coding for the stimulus [5, 24]. The estimated regression coefficient (i.e. beta) was taken to present the stimulus response, and betas from all voxels were combined to form a data matrix with columns of voxels and rows of stimuli responses of each voxel. This data matrix was then used as the input data in our MVPA analysis.

### MVPA analysis

To examine multi-class categorization of auditory objects, we followed the general framework of MVPA. A popular MVPA algorithm SVM-RFE was presented. Then the multi-class extension of SVM-RFE was introduced. In addition, we considered several other support vector machine (SVM) based approaches and their variants.

### General framework of MVPA

Typical fMRI applications of MVPA require two basic stages, model training and model testing [10, 25]. In the training stage, the MVPA model is built by learning a functional relationship between brain response patterns and mental states (experimental conditions) using the training data set. Experimental conditions are usually labeled as discrete values such as one or negative one for binary outcomes. In the testing stage, the newly trained MVPA model is used to classify the experimental conditions of an independent data set (test data) based on the brain patterns. The model performance is evaluated in terms of classification accuracy.

The fMRI data is split into a training data set given by a matrix **X** ϵ **R** ^*n*×*p*^ and a testing data set given by a matrix $\tilde{X}\in R^{m\times p}$, with corresponding labels **Y** ϵ **R** ^*n*×*1*^ and $\tilde{Y}\in R^{m\times 1}$ indicating respective experimental conditions. In the context of classification of fMRI responses, *n* denotes the training sample size, *m* denotes the testing sample size, and *p* denotes the number of voxels. The MVPA model first learns a decision function (or discriminant function), denoted as *f* **(X)**, that is a scalar function of the input brain response patterns **X**. In the case of linear classification of fMRI responses, new patterns $\tilde{X}$ are classified according to the sign of the decision function:

$$f({\tilde{x}}_{i})=w^{\text{T}}{\tilde{x}}_{i}+b,$$ (1)

Where **W** ^T^ is a 1× *p* weight vector and *b* is a bias term or threshold weight. The decision rule for a binary classifier is:

$$\begin{array}{cc} & {\tilde{y}}_{i}=1\;,{\tilde{x}}_{i}\in \text{class}(+)\;\text{if}\;f({\tilde{x}}_{i})>0,\\ & {\tilde{y}}_{i}=-1\;,{\tilde{x}}_{i}\in \text{class}(-)\;\text{if}\;f({\tilde{x}}_{i})<0, \end{array}$$ (2)

### SVM-RFE

SVM-RFE is a popular MVPA model which can be used to identify brain response patterns through training and testing a binary classifier. It has been extensively evaluated through both simulated and real fMRI data [1, 5, 24, 26]. This algorithm can effectively reduce the dimensionality of fMRI dataset by iteratively eliminating voxels with the smallest rank under a certain ranking criterion [27]. This algorithm proceeds with two sequential steps iteratively including classifier construction (support vector machine) and recursive feature elimination.

SVMs are presently one of the best-known classification techniques with computational advantages. Intuitively, an SVM searches for a hyperplane, to which the distance from the closest samples of each of two classes is maximized [28]. The decision rule of linear binary SVM follows the formula (2). The mechanism of SVMs is to minimize the following optimization problem:

Minimize $\text{J}(w)=\frac{1}{2}{\left\|w\right\|}^{2}+\text{C}\sum_{i=1}^{n}\xi_{i}$,
Subject to *y*_i_[**w** ^T^ **x**_i_ + b] ≥ 1- ξ_*i*_, ξ_*i*_ ≥ 0, *i* = 1, …, *n*.

Here, C is a predefined parameter for balancing training accuracy and model generalization. Often there does not exist a hyperlane that can perfectly split two classes. Therefore, misclassification is allowed during model training. Slack variables ξ_*i*_ are introduced to measure the degree of misclassification of the data **X**_*i*_.

Recursive feature elimination (RFE) is an iterative procedure for backward voxel elimination. It is commonly believed that in fMRI studies only parts of the brain are involved in certain information perception, thus MVPA models aim to identify a subset of voxels that can efficiently and effectively represent different processes of the stimulus of interest. RFE is such a voxel elimination strategy and can be summarized as below:

1. Train the classifier (optimize the weights *W_i_* with respect to **J(W)**)
2. Compute the discriminative weights (the ranking criterion) for all features (*W_i_* ^2^)
3. Remove the features with the smallest ranks.

### Multi-class SVM-RFE

SVM-RFE was originally designed for binary classification. In the present study, we wish to build a multi-class classification model for decoding multiple sound categories simultaneously based on the brain activities in the auditory cortex of the temporal lobe. Hence, after data pre-processing using AFNI, we applied an extension of SVM-RFE, namely multi-class SVM-RFE (MSVM-RFE), for decoding multiple sound categories [29].

We considered all voxels in the temporal lobe from both hemispheres to form initial multi-voxel patterns. The voxelwise stimulus response was estimated by a GLM where the stimulus was coded as one predictor. For this analysis, stimulus responses were estimated for each of seven functional runs separately.

The entire data set was split into two parts, a training set and a test set. For each splitting, data from the training set was used to train a classifier to compute voxel discriminative weights, and then data from the independent test set was used to test the trained classifier for evaluation of classification accuracy. By convention [27], half of the voxels with lower discriminative weights were eliminated in the RFE step in each iteration. Within the training step, cross validation was used to find the optimal number of voxels to be included in the model which yielded the smallest cross validation error. For within-subject analysis, the classifier was trained for each subject separately, with data from five functional runs as the training set and the other two runs as the test set. The process was repeated five times, each with a random choice of data splitting on the seven functional runs. For across-subject analysis, data from each individual subject was left out once as a test set while data from all other subjects was treated as a training set. Classification accuracies calculated from test sets were averaged over all repetitions. The selected voxels were mapped back to the brain template as a discriminative map for each classification.

To investigate the brain’s representation of sound categories, we constructed sound category-selective maps. Similar to univariate contrast analysis, category-selective maps are often defined based on the results of binary classification algorithms [3]. For example, the English-selective map can be defined as the conjunction of six discriminative maps related to the English category, which are obtained through the binary classifications of English versus each of the other six sound categories. In this study, we made use of multi-class classification to find category-selective maps. For example, the English-selective map is defined as the significant remaining voxels in a comparison between 7- (all) and 6-class (without English) classifiers. We combined the selected voxels from all subjects and mapped these voxels, which consistently survived in the RFE selection for more than one repetition and more than one subject, back to the brain template as category-selective maps.

### Other MVPA methods

As SVM-RFE is one of many possible support vector machines, we also examined two other SVM models including L2-regularized L1-loss support vector classification [30] and Crammer-Singer multi-class SVM [30]. Although an SVM is often understood as a method of searching the maximum-margin hyperplane, it can also be formulated as a hinge loss function plus a L2-norm regulation term. The so-called L2-regularized L1-loss support vector classification is a support vector machine with a L2-norm regularization scheme and a L1-norm loss function. The Crammer-Singer multi-class SVM is a support vector machine that can classify multiple classes without voxel selection ability [31]. Since fMRI data is high dimensional, we also considered the application of penalized linear discriminant analysis (PLDA), one advanced statistical method commonly used in high dimensional data analysis [32]. Linear discriminant analysis is a classical method for dimension reduction and classification, which aims to maximize the ratio of between-class variance over within-class variance. PLDA is a general approach for penalizing the discriminant vectors in Fisher’s linear discriminant analysis in a way that leads to greater interpretability. The results from these methods will be compared in the next section.

## Results

### Multi-class classification within subjects

**Fig 1:**
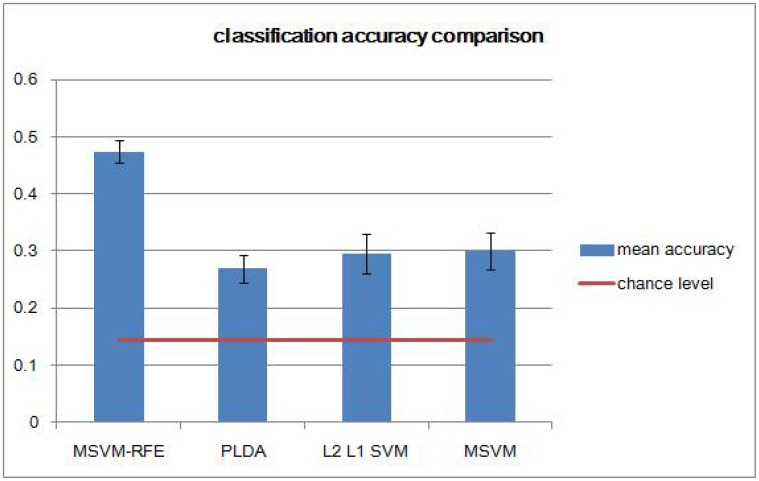
Comparison of seven-sound category classification accuracies obtained by four different MVPA models. Error bars represent one standard deviation of the mean accuracy.

In general, multi-class prediction is much harder than solving binary prediction [29]. This problem becomes even more challenging in fMRI data due to the large number of voxels and relatively small number of samples for each stimuli and category. We first explored the classification performance of several multi-class MVPA models including MSVM-RFE, PLDA, L2-regularized L1-loss support vector classification, and Crammer-Singer multi-class SVM. A 7-class classifier was built by using each of these statistical methods with five repetitions. As shown in Fig. 1, MSVM-RFE achieved classification accuracies well above the other three models. The horizontal line marks the chance level. It should be noted that there are many more available MVPA models in the literature. For our specific problem, the MSVM-RFE model is chosen for analyzing the fMRI data.

**Fig 2:**
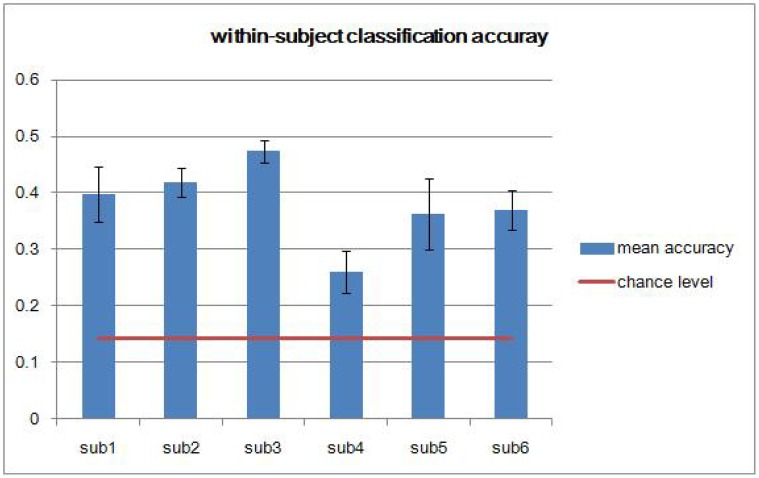
Classification accuracies for seven-sound category classifiers built for each subject separately using MSVM-RFE. Error bars represent one standard deviation of the mean accuracy.

To estimate and decode the distinct activation patterns elicited by different sound categories, we trained and tested the MSVM-RFE model for decoding all seven sound categories simultaneously within each subject. Fig. 2 shows the classification accuracies for the within-subject analysis with the chance level marked by the horizontal line. The MSVM-RFE model was able to learn the functional relation between the sound categories and the corresponding evoked spatial patterns, and achieved classification accuracies for each subject significantly above the chance level (p = 3 × 10^-4^ for subject 1, p < 1 × 10^-4^ for subjects 2, and 3, p = 2.6 × 10^-3^ for subject 4, p = 1.7 × 10^-3^ for subject 5, and p = 1 × 10^-4^ for subject 6, one sample two-sided t test, n = 5). This demonstrates the feasibility of decoding multiple sound categories within subjects. From Fig. 2, we also observed some indication of variability in classification performance across subjects.

**Fig 3:**
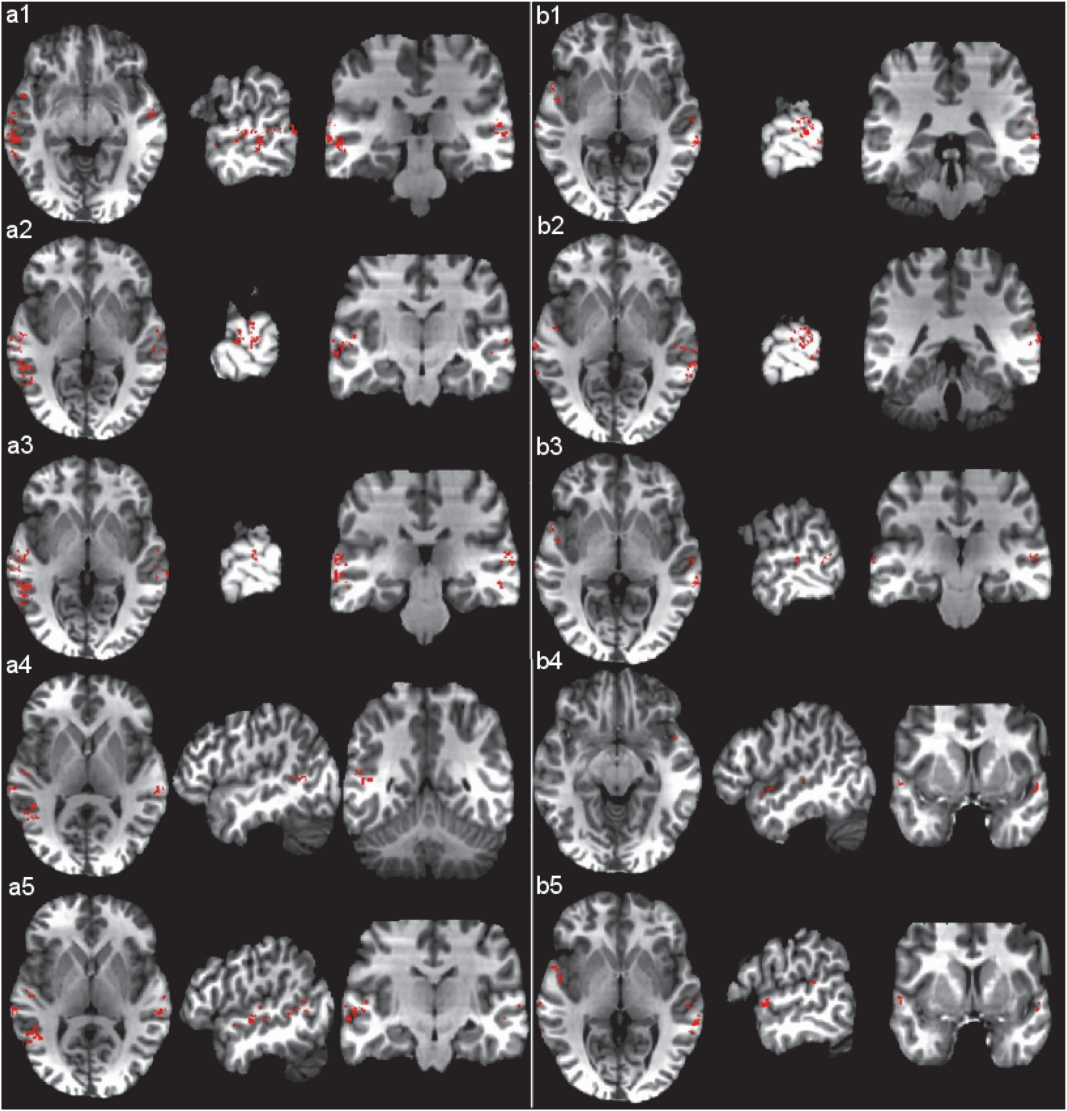
Selective brain maps for (a) English and (b) Music categories based on MSVM-RFE classification results. Panels (a1) – (a4) show the four largest clusters from voxels selected for the English category. Panel (a5) shows the cluster on the left Heschl’s gyrus. Panels (b1) – (b5) show the five largest clusters from voxels selected for the Music category. For each panel, the axial, sagittal and coronal slices are centered at the corresponding cluster. The left side of the brain is on the left side of the figure.

To further explore the brain’s representation of sound categories, we constructed sound category-selective maps. Fig. 3 shows selective brain maps for the English and Music categories separately, both of which revealed distributed patterns of brain activity in the superior temporal gyrus (STG) and the middle temporal gyrus (MTG). The English-selective map which differentiated English from the other six sound categories was widely distributed bilaterally in the STG with the largest cluster (corresponding approximately to MNI coordinate -58, -30, -9) in the left MTG. Panels (a1) – (a4) in Fig. 3 show the four largest clusters from voxels selected for the English category in the left MTG (-58, -30, -9), the left STG (-65, -16, 4), the right STG (64, -27, 2) and the left MTG (-46, -53, 6) correspondingly. Panel (a5) shows the cluster on left Heschl’s gyrus (-49, -16, 7). As shown in Panels (b1) – (b5), the five largest clusters for the music-selective map were found in the right MTG (64, -37, -1), the right MTG (65, -41, 2), the right STG (58, -24, 0), the right STG (47, 11, -9) and the left STG (-55, 9, 3), and the discriminative patterns for music were widely distributed bilaterally in the superior temporal regions. In the brain decoding literature, a distributed pattern of brain responses is not clearly defined. In the domain of visual perception, the brain’s representation of visual-object categories was found to be widely distributed, reflected by a distinct pattern of response across a wide expanse of cortex [14]. The same type of category specific distributed pattern was observed in our study as well as several other brain decoding studies in the auditory domain [2, 5]. Our results indicate that representations of sound categories are widely distributed along the temporal cortex. This is in agreement with previous studies, indicating that information in the spatially distributed patterns may reflect a more abstract perceptual level of representation of sound categories [1, 2].

### Multi-class classification across subjects

**Fig 4:**
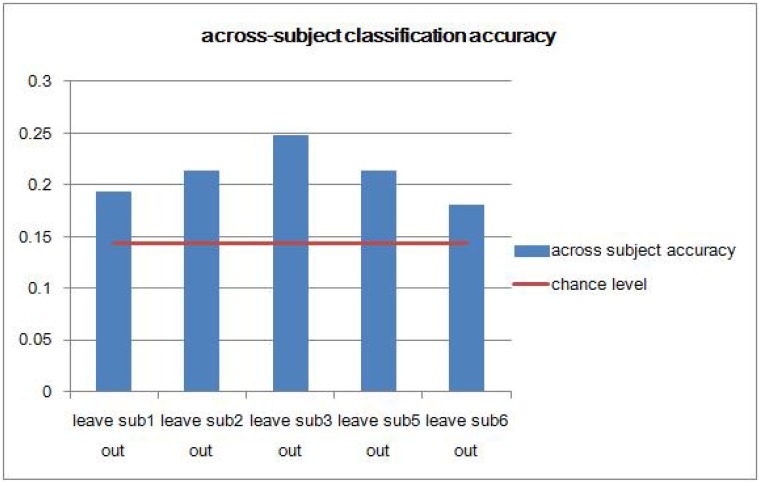
Classification accuracies across subjects for all seven sound categories using MSVM-RFE. Based on within-subject analysis results, the data from subject 4 was not included for the across-subject analysis. “leave sub1 out” means leaving subject 1 out. That means the model is trained on data from the 2nd, 3rd, 5th, and 6th subjects and is tested on data from the 1st subject.

To investigate whether sound category-selective activity patterns are consistent across subjects and how results from pattern analysis are generalized across individuals, we performed the across-subject analysis. Because the within-subject classification accuracies obtained from subject 4 were significantly lower than accuracies obtained from any other subject (Fig. 2), we excluded this subject from further analysis. One way to test whether there are consistent brain response patterns across subjects is to train a classifier on data from multiple individuals and test the classifier on data from a new individual [20]. Hence, in the across-subject analysis, we iteratively left out the complete data of one subject. The model for classifying seven sound categories simultaneously was trained on four subjects and was tested on the left out subject. From Fig. 4, we can see the prediction performance is significantly above chance (p = 7.3 × 10^-3^, one sample two-sided t test, n = 5), which demonstrates the feasibility of decoding multiple sound categories across subjects. This indicates that neural activity patterns in the human temporal cortex reflect the categorical content of sounds and that these patterns share features across subjects. The horizontal line represents the accuracy achieved from guessing. By comparing Fig. 2 and Fig. 4, we can see that across-subject classification accuracies are significantly lower than within-subject classification accuracies (p < 1 × 10^-4^, two sample two-sided t test, *n*_1_ = 25, *n*_2_ = 5), indicating that a significant portion of the neural representation of the sound categories is specific to the individual. This suggests that the across-subject variation affects classification performance more than within-subject variation. Compared with the within-subject analysis, across-subject generalization is more challenging due to the additional inter-subject variability. The success of decoding strategies will also be dependent on whether it is possible to identify functionally matching brain regions in different subjects.

### Multi-class classification on averaged items and subjects

**Fig 5:**
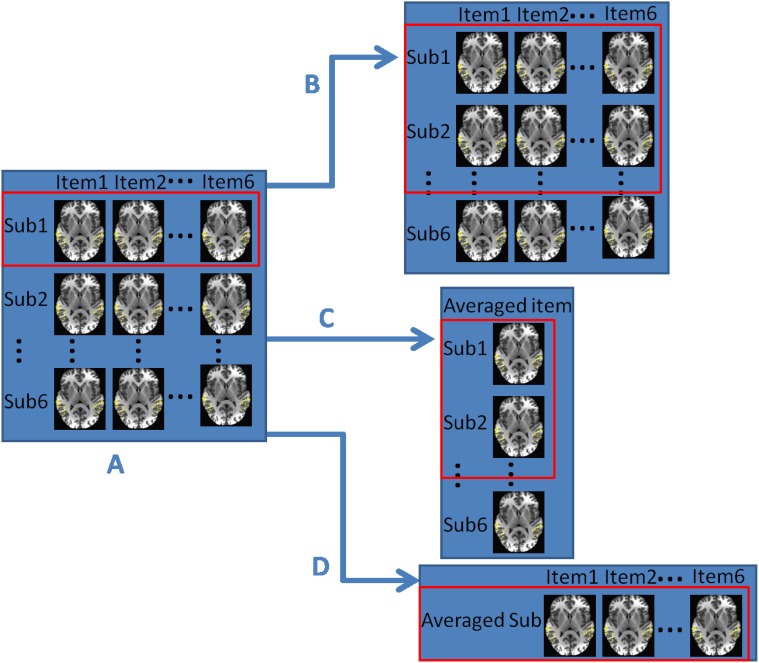
The relationship chart of four different fMRI data analysis cases. (A) Case WithinSub: Classifiers are trained and tested for each subject separately; (B) Case AcrossSub: Classifiers are built across subjects, that is, leaving out the complete data of one subject for testing and using the data from all other subjects for training; (C) Case AvgItem: fMRI data are averaged over items and classifiers are built across subjects on the averaged data; (D) Case AvgSub: fMRI data are averaged over subjects and classifiers are built for this “averaged subject”. For each fMRI data analysis case, if a red rectangle contains only one subject, it indicates a within-subject analysis. Otherwise, the analysis is done across subjects.

Results from multi-class classification within and across subjects motivated us to further investigate effects of averaging over items and over subjects on classifier performance. All four scenarios and their relationship are summarized in Fig. 5.

The within- and across- subject analysis described in the previous two subsections are called Cases WithinSub and AcrossSub, shown as Parts A and B of Fig. 5. To study the generalizability across different sound exemplars (items) within the same sound category, we averaged the fMRI images over items (Case AvgItem), leading to fMRI data on an “averaged item”. Averaging over items in Case WithinSub leads to Case AvgItem (Part C of Fig. 5). Further, we averaged the fMRI images over subjects to investigate the effect of averaging over subjects (Case AvgSub), leading to fMRI data on an “averaged subject”. Averaging over subjects in Case WithinSub leads to Case AvgSub (Part D of Fig. 5). For Cases WithinSub and AvgSub, the red rectangle in Fig. 5 only includes one subject, which indicates the classifier is within-subject. For Cases AcrossSub and AvgItem, the red rectangle includes multiple subjects, which indicates that the classifier is trained on data from a subgroup of the subjects and is then tested on data from a subject that is not part of that subgroup (Fig. 5).

**Fig 6:**
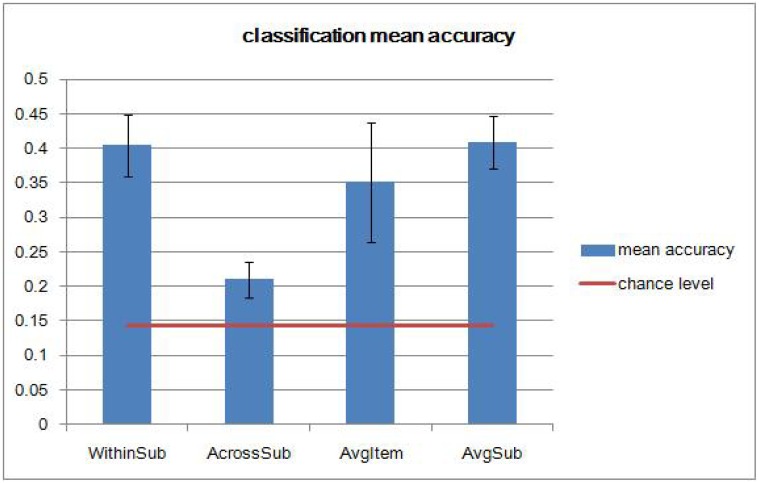
Mean accuracy comparison of seven-sound category classifiers under four different cases using MSVM-RFE. Error bars represent one standard deviation of the mean accuracy.

As described above, seven-sound category classifiers were built for each case. Averaged classification accuracies under each case are shown in Fig. 6. Classification accuracies under Case WithinSub are significantly higher than accuracies under Case AcrossSub. This suggests the across-subject variation affects classification performance more than the within-subject variation. More intriguingly, it is found that classification accuracies under Case AvgItem are significantly higher than accuracies under Case AcrossSub (p = 0.0196, two sample two-sided t test, *n*_1_ = 5, *n*_2_ = 5)， though models built for these two cases are both across-subject multi-class classifiers. This suggests that averaging over items helps to improve classifier performance. Perceptual categorization of incoming sounds requires perceptual invariance of sets of sounds classified as belong to the same category. By averaging the fMRI images over items, we reduce the irrelevant variations between different items of the same sound category and in turn increase the proportion of signals relevant to sound categorization. This is reasonable in the sense that data with more repetitions on items is more stable and suffers less from noise. By comparing Case AvgSub and Case WithinSub, as expected, classification performance on the “averaged subject” is close to the average of classification performance on individual subjects.

**Fig 7:**
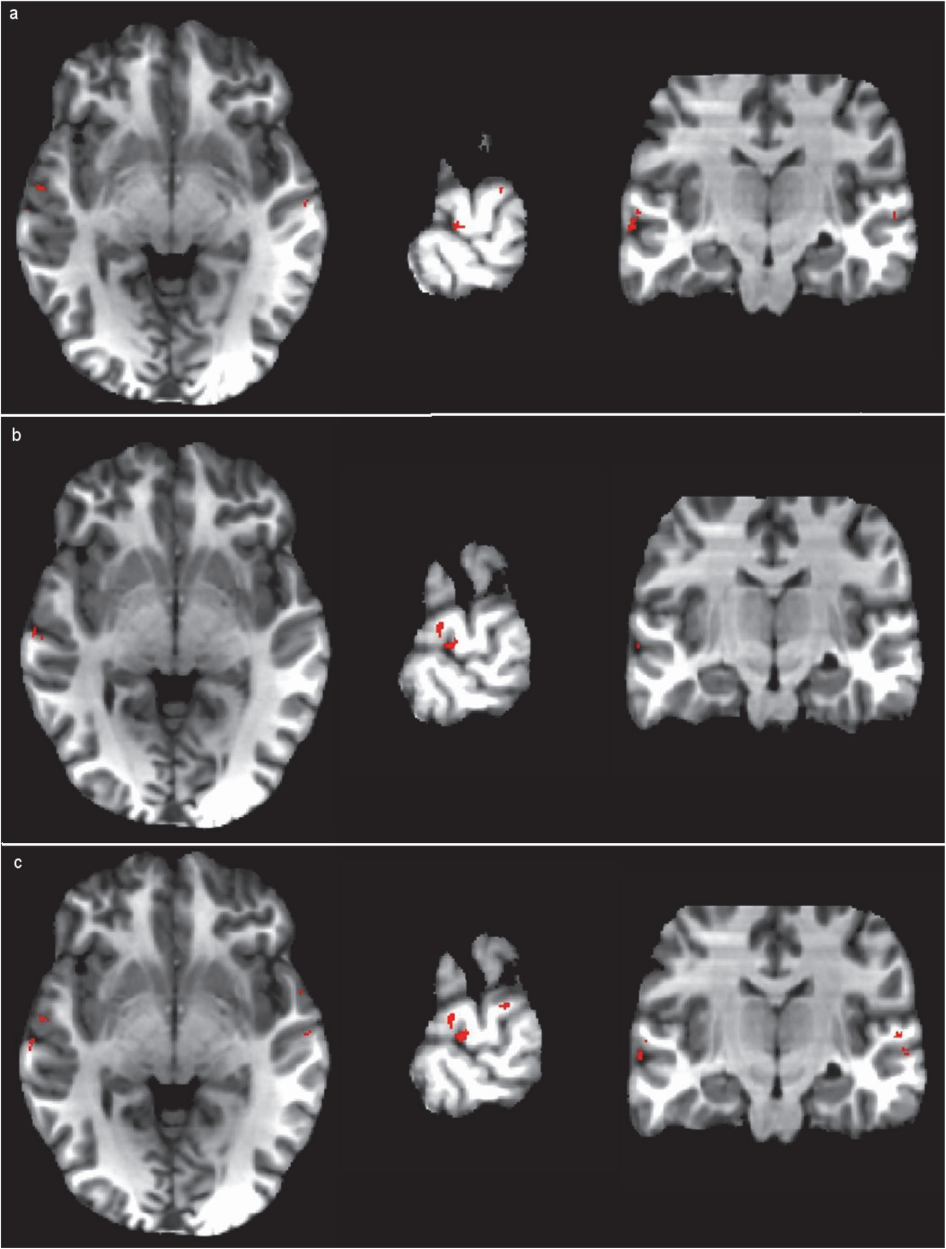
Conjunction brain maps for common voxels between Cases WithinSub and each of the other three. Brain maps of the largest clusters formed by selected voxels that are common between Cases WithinSub and AvgSub, Cases WithinSub and AvgItem, and Cases WithinSub and AcrossSub are shown in parts (a), (b), and (c) respectively. The left side of the brain is on the left side of the figure.

To further understand the relationship between the four cases, we performed conjunction brain mapping of the voxels selected under Cases WithinSub and AvgSub, Cases WithinSub and AvgItem, and Cases WithinSub and AcrossSub. Since Case WithinSub (within-subject analysis) is the most common way for doing fMRI data analysis, we compared the selected voxels that are common and different between Case WithinSub and each of the other three cases. Again, the revealed patterns of brain activity are distributed. Despite these differences, we found that, more interestingly, the largest clusters (corresponding approximately to MNI coordinate (-65, -15, -6), (-63, -13, -3), and (-63, -14, -4) in Fig. 7 parts (a), (b), and (c) respectively) formed by selected voxels that are common between Case WithinSub and each of the other three cases are approximately at the same brain locations, indicating that classification in these cases appear to rely on similar brain areas (Fig. 7). Though accuracies do vary among the four cases due to subject variation and other factors, the common voxels revealed in the conjunction brain maps in Fig. 7 seem to be essential for successful classification. This also suggests that multi-voxel patterns of BOLD responses evoked by auditory stimuli are informative and can be used to decode multiple sound categories not only within but across subjects.

## Discussion

### Item and subject variations

In this study, both item and subject variations are found to have significant effects on model decoding performance.

One challenging question is the extent to which MVPA decoding strategies might be generalized across different exemplars of the same sound category [9]. Perception of sound categories requires perceptual invariance of sets of sounds classified as belonging to the same category, resulting in a more abstract representation of sound similarity [1]. Successful decoding strategies should be able to increase the process of relevant information and decrease the process of irrelevant information. This necessitates a certain degree of flexibility in MVPA modeling so that the model ignores irrelevant variations between different items of the same sound category and selects similar features as the basis of abstract representations of sound categories. However, the ability of MVPA models to identify a distinctive brain response pattern corresponding to one sound category depends on the selection of sound exemplars as belonging to this category. If a sound category is well represented by the chosen exemplars, the generalization across different instances of the same sound category is facilitated. By averaging the fMRI images over items, we reduce the irrelevant variations between different items of the same sound category and in turn increase the proportion of signals relevant to sound categorization. The averaged item seems to possess the greatest number of properties defining this category, and hence could be considered as the most typical exemplar of this class. Similar to the exemplar theory in the visual categorization literature, categorization is based on a comparison of the stimulus with all previously categorized exemplars of all categories [33]. Having more sound exemplars in one sound category may help to improve model decoding performance.

The same-category sounds, while highly variable, may show considerable acoustic similarity compared with between-category sounds. In this scenario, MVPA models could potentially be keying on physical rather than categorical differences during training. Similar to previous studies [2, 3], all stimuli in this study were resampled to the same sampling rate and normalized to the same intensity level and fundamental frequency. However, we cannot completely rule out the role of acoustic variables because semantic and acoustic categories could be correlated. Perfect normalization of acoustic differences would produce a set of identical stimuli [3]. Hence, some acoustic variability across categories is expected. The relationships between the sound categories and the acoustic features are complex. Previous work has established that people categorize sound samples on the basis of semantic features rather than strictly perceptual ones, which means that semantic properties are not directly reducible to causal physical parameters [34]. Although the perceived similarities among environmental sounds are strongly determined by acoustic features, sound categorization is less well predicted by acoustic features [35].

Another challenging question is the decoding model generalizability across subjects. It is known that there is not always precise spatial correspondence between homologous functional locations in different individual brains, even when advanced alignment procedures are used [9]. Such subject variation will necessarily obscure any across-subject generalization. Though there are subject variations in anatomy and methodological difficulties in inter-subject co-registration, neural similarities arose in terms of the locations and activation amplitudes of voxels utilized by the classifier to identify the category of a stimulus [36]. There are now a growing number of studies in showing that MVPA can uncover invariant patterns of neural activity across subjects [20, 37, 38].

### Localized versus distributed representation of sound categories

In the literature of fMRI studies on the brain’s representations of sound categories, previous models suggest a hierarchical processing of auditory categories in the auditory cortices. In these models, the superior temporal cortex is organized in specialized areas among which the neural processing of a sound proceeds from the analysis of its low-level physical constituents to higher perceptual dimensions. Several groups have used univariate approaches and identified selective regions in the auditory cortex that potentially account for sound category differentiation [3, 4, 39]. However, Staeren *et al*. found that representations of sound categories in the superior temporal cortex are widely distributed [2]. In our study, sound category-selective brain maps were identified based on multi-class classification and revealed distributed patterns of brain activity along the temporal cortex. This is in agreement with previous studies, indicating that information in the spatially distributed patterns may reflect a more abstract perceptual level of representation of sound categories [1, 2]. This suggests that the brain’s representations of sound categories could emerge from the joint encoding of information occurring in a set of areas associated with not only higher-level but also lower-level auditory processing. Future research should consider the interaction between semantic and physical properties of sound categories and their weighting of the properties in sound encoding, including how sounds within the same semantic category with both different or the same acoustic features are represented, and whether focal and distributed representations are related to categorical hierarchies.

The localized and distributed views of sound categorization may not be completely incompatible with each other. It is possible for one area to play a dominant role, while several different areas offering complementary support. Kumar *et al*. argued the possibility of incorporating the role of a pitch center within a distributed system for human brain pitch representation [40]. Similar arguments may be made for sound category representation in the human brain.

We acknowledge that the sample size in our current study is relatively small, nevertheless this sample size is consistent with similar studies reported in the literature [1, 2, 5, 19, 20]. We believe our study demonstrates that it is possible to generalize sound categorization using fMRI measures from one listener to another. However, we make no claim that the data we obtained could be generalized to all listeners in the population. In addition, we acknowledge that our results are limited to the seven chosen sound categories and further research is needed to explore how other sound categories are represented in the brain. Nevertheless, we included a number of sound categories that can be found in the natural world. Thus, we believe that our results speak to fundamental principles about sound category encoding in the brain.

## Summary

In this study, we investigated the representation and processing of sound categories within the human temporal cortex using high resolution fMRI and MVPA methods. Critically, we examined the benefits of using MSVM-RFE to decode multiple sound categories simultaneously. Reliable pattern classification is challenging due to multiple sound categories, the high dimensionality of the dataset, and small sample sizes for each stimuli and category.

We have shown that for all classifications the model MSVM-RFE was able to learn the functional relation between the multiple sound categories and the corresponding evoked spatial patterns and classify the unlabeled sound-evoked patterns significantly above chance. This indicates the feasibility of decoding multiple sound categories not only within but across subjects. Sound category-selective brain maps were identified based on multi-class classification and revealed distributed patterns of brain activity. Our across-subject analysis reveals that neural activity patterns in the human temporal cortex reflect the categorical content of sounds and that these patterns share features across subjects. However, the across-subject variation affects classification performance more than within-subject variation, as the across-subject analysis has significantly lower classification accuracies than the within-subject analysis. Further, we show that by averaging the fMRI images over items, the across-subject classification performance can be significantly improved. After averaging the fMRI images over items, the irrelevant variations between different items of the same sound category are reduced and in turn the proportion of signals relevant to sound categorization is increased. This necessitates a certain degree of flexibility in MVPA modeling so that the model ignores irrelevant variations between different instances of the same sound category and selects similar features as the basis of abstract representations of sound categories. In addition, this study improves our understanding of an important and unresolved question, i.e., the level to which MVPA decoding models can be generalized across multiple sound categories, across different subjects, and across different sound exemplars within the same category.
